# Exploring the Birthday Week Effect on Hand, Foot, and Mouth Disease in Yunnan Province, China, From 2008 to 2022: Surveillance Data Analysis

**DOI:** 10.2196/59237

**Published:** 2024-09-09

**Authors:** Pei Jiang, Xiangyu Yan, Tongjian Cai, Longxin Huang, Zhenzhong Liu, Linhui Hao, Tian Huang, Haijun Yang, Min Xu, Wenhui Shi, Tiejun Shui

**Affiliations:** 1 School of Public Health, North Sichuan Medical College, Nanchong, China; 2 School of Disaster and Emergency Medicine, Tianjin University, Tianjin, China; 3 Department of Epidemiology, College of Preventive Medicine, Army Medical University (Third Military Medical University), Chongqing, China; 4 School of Clinical Medicine, North Sichuan Medical College, Nanchong, China; 5 Yunnan Center for Disease Control and Prevention, Xianghe Street #1177, Chenggong District, Kunming, 650050, China, 86 13987165649; 6 Yan'An Hospital of Kunming City, Kunming, China; 7 Hospital of Xi Zang Medicine, Lhasa, China; 8 Lanke Medical Technology Nanjing Research Institution, Nanjing, China

**Keywords:** hand, foot, and mouth disease, birthday week effect, infants, children, China, surveillance, HFMD

## Abstract

**Background:**

Hand, foot, and mouth disease (HFMD) is a notable infectious disease predominantly affecting infants and children worldwide. Previous studies on HFMD have primarily focused on natural patterns, such as seasonality, but research on the influence of important social time points is lacking. Several studies have indicated correlations between birthdays and certain disease outcomes.

**Objective:**

This study aimed to explore the association between birthdays and HFMD.

**Methods:**

Surveillance data on HFMD from 2008 to 2022 in Yunnan Province, China, were collected. We defined the period from 6 days before the birthday to the exact birthday as the “birthday week.” The effect of the birthday week was measured by the proportion of cases occurring during this period, termed the “birthday week proportion.” We conducted subgroup analyses to present the birthday week proportions across sexes, age groups, months of birth, and reporting years. Additionally, we used a modified Poisson regression model to identify conditional subgroups more likely to contract HFMD during the birthday week.

**Results:**

Among the 973,410 cases in total, 116,976 (12.02%) occurred during the birthday week, which is 6.27 times the average weekly proportion (7/365, 1.92%). While the birthday week proportions were similar between male and female individuals (68,849/564,725, 12.19% vs 48,127/408,685, 11.78%; *χ*^2^_1_=153.25, *P*<.001), significant differences were observed among different age groups (*χ*^2^_3_=47,145, *P*<.001) and months of birth (*χ*^2^_11_=16,942, *P*<.001). Compared to other age groups, infants aged 0‐1 year had the highest birthday week proportion (30,539/90,709, 33.67%), which is 17.57 times the average weekly proportion. Compared to other months, patients born from April to July and from October to December, the peak months of the HFMD epidemic, had higher birthday week proportions. Additionally, a decreasing trend in birthday week proportions from 2008 to 2022 was observed, dropping from 33.74% (3914/11,600) to 2.77% (2254/81,372; Cochran-Armitage trend test: *Z*=−102.53, *P*<.001). The results of the modified Poisson regression model further supported the subgroup analyses findings. Compared with children aged >7 years, infants aged 0‐1 year were more likely to contract HFMD during the birthday week (relative risk 1.182, 95% CI 1.177‐1.185; *P*<.001). Those born during peak epidemic months exhibited a higher propensity for contracting HFMD during their birthday week. Compared with January, the highest relative risk was observed in May (1.087, 95% CI 1.084‐1.090; *P*<.001).

**Conclusions:**

This study identified a novel “birthday week effect” of HFMD, particularly notable for infants approaching their first birthday and those born during peak epidemic months. Improvements in surveillance quality may explain the declining trend of the birthday week effect over the years. Higher exposure risk during the birthday period and potential biological mechanisms might also account for this phenomenon. Raising public awareness of the heightened risk during the birthday week could benefit HFMD prevention and control.

## Introduction

Hand, foot, and mouth disease (HFMD) is a globally prevalent infectious disease caused by various enteroviruses, including coxsackievirus A16 and enterovirus 71 [1]. While most patients exhibit benign and self-limiting clinical symptoms, some may develop severe complications, such as central nervous system damage, cardiopulmonary failure, and even death [2]. Over the past decades, HFMD outbreaks have occurred frequently in the Asia-Pacific region [3]. In 2008, HFMD was classified as a Class C infectious disease for surveillance in China, with annual incidence number exceeding 1 million [4,5]. Among HFMD cases, children <5 years old are the most affected, with those aged between 6 months and 2 years exhibiting the highest incidence and severe illness rates [6].

HFMD is predominantly transmitted through the fecal-oral route via direct person-to-person contact, as well as indirect contact with surfaces and objects contaminated with excretions, such as oral secretions and vesicular fluid from infected individuals [7]. HFMD is highly contagious, and previous studies have reported numerous infections associated with high concentrations of people, such as at family gatherings and in kindergartens [8-10].

Previous studies have shown that the incidence of HFMD is influenced by various factors, including region and season [6]. HFMD exhibits seasonality. In South China, HFMD has 2 peaks: the main peak from April to July and a secondary peak from October to December [11,12]. This seasonality is related to the increased prevalence of HFMD viruses in the natural environment during these periods, influenced by climatic and meteorological changes [13,14]. In addition to natural factors, diseases occurrence is also related to social factors, with birthday being an important social time point. In recent years, several studies have reported positive associations between birthdays and various health and disease outcomes, including suicide [15,16], average excess death rate [17], mortality after surgery (if performed on the surgeon’s birthday) [18], vascular events [19], medical emergency department attendance [20], and COVID-19 infections [21]. However, no studies have examined the relationship between birthdays and HFMD, an infectious disease that primarily affects infants and children.

In China, the national infectious disease surveillance system records the date of onset and birth date of the patient, providing an opportunity to study the birthday rhythm phenomenon in major infectious diseases such as HFMD. Previous studies on HFMD have treated age merely as a basic demographic characteristic, reporting incidence and other measures of interest by age groups according to convention [22,23]. This approach misses the opportunity to show the possible birthday rhythm phenomenon by not presenting age in days. In this study, we used HFMD surveillance data from 2008 to 2022 in Yunnan Province, China, to explore the birthday rhythm phenomenon of HFMD.

## Methods

### Study Setting

Yunnan Province is situated on China’s southwestern border, between longitudes 97.31°E and 106.11°E and between latitudes 21.80°N and 29.15°N, within the Greater Mekong Subregion [24]. It has a population of approximately 47 million and covers 394,000 km^2^ (as of 2021) [25]. Due to its unique geographical and climatic environment, Yunnan Province has consistently exhibited a high incidence of infectious diseases, including HFMD [22]. Consequently, the study of infectious diseases in Yunnan Province is of great significance.

### Data Sources

HFMD surveillance data from January 1, 2008, to December 31, 2022, were collected from the National Surveillance of Notifiable Infectious Disease Program (NSNIDP) established by the China Centers for Disease Control and Prevention (CDC). Since 2008, HFMD has been classified as a notifiable infectious disease, requiring reporting to the NSNIDP [4]. The system recorded the sex, date of birth, and date of onset of HFMD cases.

### Statistics Analysis

In this study, “age in days” was calculated for each HFMD case to serve as the basis for subsequent statistics analyses. The “age in days” was calculated as follows:

*Age in days = the difference in years between the onset date and last birthday × 365 + the difference in days between the onset date and last birthday*

where “last birthday” refers to the date of the last birthday before the HFMD onset. We presented the number of HFMD cases with “age in days” as the abscissa in plotting. The “age in days” calculation method differs from the simpler and more common way (the difference in days between the onset date and birth date) because our method is more accurate and convenient for plotting. By using 365 days as a fixed period, we can determine the floor function of “age in years” and the number of days deviating from the birthday. In contrast, the other calculation method is more complicated as it requires accounting for the number of leap years between the birth date and onset date, especially for older individuals.

We defined the “birthday week” as the period spanning from 6 days before the birthday to the exact birthday (namely, the period in which the difference in days between last birthday and onset date was from 359 to 365 days). We also calculated the proportion of cases occurring during the birthday week, termed the “birthday week proportion,” to quantitatively access the birthday week effect.

We calculated the birthday week proportions across different sex and age groups (0‐1 y, 1‐3 y, 3‐7 y, and >7 y). We also conducted analyses stratified by months of birth and reporting years. The classifications of age groups were based on the activity characteristics of children. Children aged 0‐3 years are usually cared for at home, with those aged 0‐1 year classified as infants. Children aged 3‐7 years usually attend kindergartens, with those aged >7 years going to school. The Pearson *χ*^2^ test was used to analyze the differences in “birthday week proportions” among different age groups, sexes, and months of birth. The Cochran-Armitage trend test was used to perform trend analyses of birthday week proportions overall and within different age groups.

In addition, we conducted a modified Poisson regression model to identify conditional subgroups by sex, age group, month of birth, and reporting year that were more likely to contract HFMD during the birthday week. In the model, the dichotomous outcome variable is “whether the incidence date falls within the birthday week” [26]. All analyses in this study were conducted using R (version 4.3.0; R Foundation for Statistical Computing).

### Ethical Considerations

The study was approved by the Research Ethics Committee of the Yunnan CDC (No. 2023‐19) with a waiver of informed consent because the data were deidentified.

## Results

### Demographic Characteristics of HFMD Cases

Between 2008 and 2022, the cumulative number of HFMD cases was 973,410, of which 564,725 (58.02%) were male and 408,685 (41.98%) were female. The male-to-female sex ratio was 1.38. In terms of age distribution, cases aged 0‐7 years accounted for 95.71% (Table 1).

**Table 1.** Cumulative HFMD^a^ cases, cases during the birthday week, and birthday week proportions by sex and age group in Yunan Province, China (2008‐2022).

Variables and age groups (years)		Total cases	Male cases	Female cases	Male-to-female sex ratio
**Cumulative cases (total cases: N=973,410; male cases: n=564,725; female cases: n=408,685), n (%)**					
	0-1	90,709 (9.32)	53,428 (9.46)	37,281 (9.12)	1.43
	1-3	485,871 (49.91)	280,236 (49.62)	205,635 (50.32)	1.36
	3-7	355,075 (36.48)	207,990 (36.83)	147,085 (35.99)	1.41
	>7	41,755 (4.29)	23,071 (4.09)	18,684 (4.57)	1.23
	Total	973,410 (100)	564,725 (100)	408,685 (100)	1.38
**Cases during the birthday week (total cases: n=116,976; male cases: n=68,849; female cases: n=48,127), n (%)**					
	0-1	30,539 (26.11)	18,061 (26.23)	12,478 (25.93)	1.45
	1-3	55,057 (47.07)	32,395 (47.05)	22,662 (47.09)	1.43
	3-7	26,856 (22.96)	15,906 (23.1)	10,950 (22.75)	1.45
	>7	4524 (3.87)	2487 (3.61)	2037 (4.23)	1.22
	Total	116,976 (100)	68,849 (100)	48,127 (100)	1.43
**Birthday week proportions, n/N (%)**					
	0-1	30,539/90,709 (33.67)	18,061/53,428 (33.80)	12,478/37,281 (33.47)	—^b^
	1-3	55,057/485,871 (11.33)	32,395/280,236 (11.56)	22,662/205,635 (11.02)	—
	3-7	26,856/355,075 (7.56)	15,906/207,990 (7.65)	10,950/147,085 (7.44)	—
	>7	4524/41,755 (10.83)	2487/23,071 (10.78)	2037/18,684 (10.90)	—
	Total	116,976/973,410 (12.02)	68,849/564,725 (12.19)	48,127/408,685 (11.78)	—

a HFMD: hand, foot, and mouth disease.

b Not applicable.

### Birthday Week Effect

#### Overview

**Figure 1.**
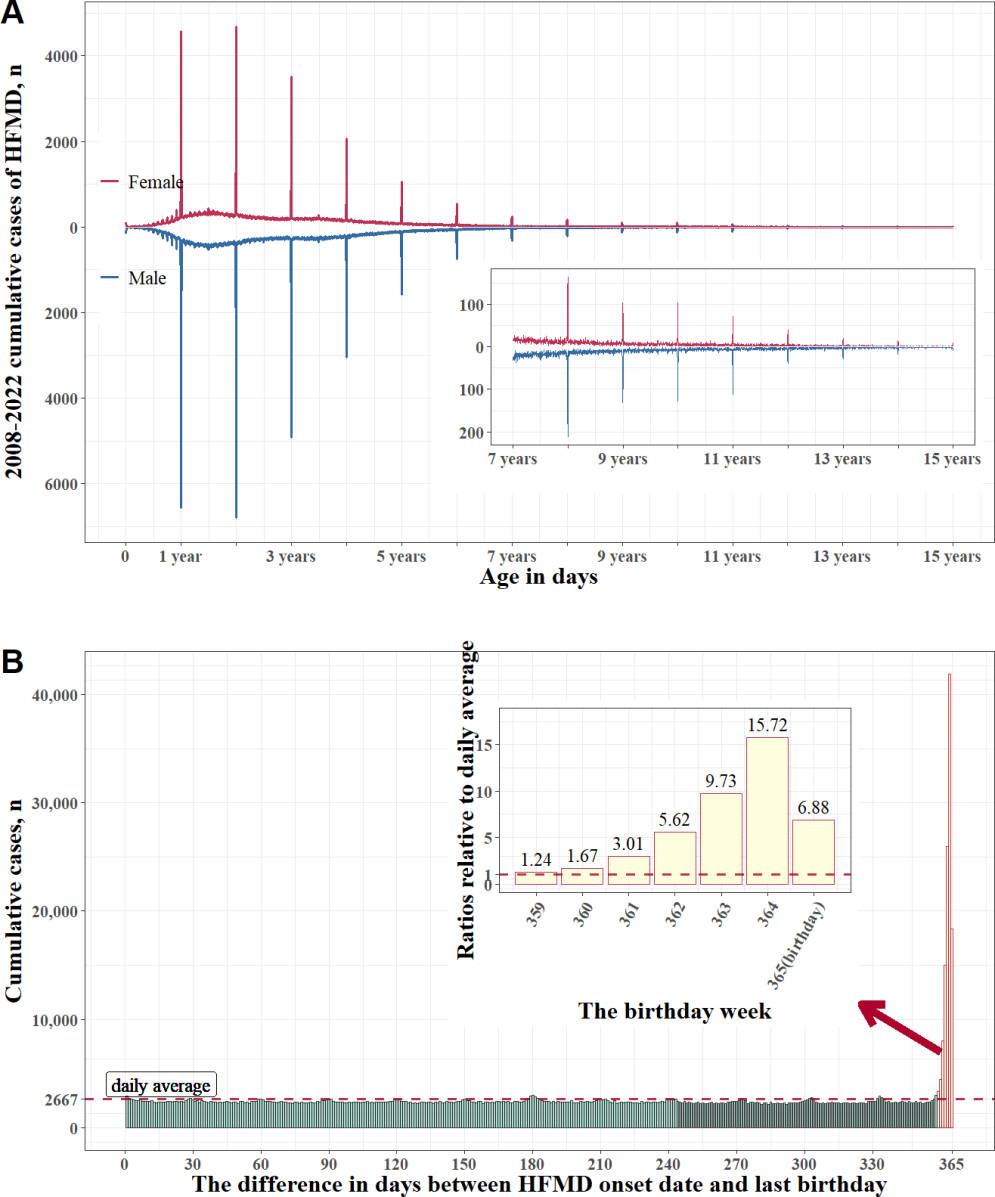
The “pulse and rhythm-type” surge in cumulative HFMD cases during the birthday week in Yunnan Province, China (2008‐2022). (A) Cumulative cases by age (measured in days) and sex among children aged 0‐15 years. (B) Cumulative cases based on the difference in days between onset date and birthday, along with ratios relative to the average daily proportion (1/365, 0.27%) for each day of the birthday week. HFMD: hand, foot, and mouth disease.

Figure 1A demonstrates an obvious “pulse and rhythm-type” surge in the cumulative HFMD cases number from 2008 to 2022 on and around birthdays for all ages, across both male and female individuals. For the 365 days in a year, on the eve of the birthday, the case numbers increased day by day as the birthday approached, peaking the day before the birthday and substantially decreasing after the birthday (Table S1 in Multimedia Appendix 1 and Figure 1B). The cumulative case numbers (and their proportions of the 973,410 total cases) from the sixth day before the birthday to the exact birthday were 3308 (0.34%); 4442 (0.46%); 8028 (0.82%); 14,982 (1.54%); 25,945 (2.67%); 41,930 (4.31%); and 18,341 (1.89%), respectively. Correspondingly, the ratios relative to the average daily proportion (1/365, 0.27%) of these 7 days were 1.24, 1.67, 3.01, 5.62, 9.73, 15.72, and 6.88, respectively. In total, the cumulative case number during the birthday week was 116,976, accounting for 12.02% of the 973,410 total cases, which is 6.27 times the average weekly proportion (7/365, 1.92%; Table S1 in Multimedia Appendix 1 and Figure 1B).

Table 1 also shows the cumulative case numbers during the birthday week and birthday week proportions across different age groups and sexes. Significant differences in birthday week proportions among different age groups were observed (*χ*^2^_3_=47,145, *P*<.001). Compared to other age groups, the infant group aged 0‐1 year had the highest birthday week proportions (30,539/90,709, 33.67%), which is 17.57 times the average weekly proportion. In the aged 1‐3 and 3‐7 years groups, birthday week proportions decreased to 11.33% (55,057/485,871) and 7.56% (26,856/355,075), respectively. For male and female groups, there was a minimal difference in overall birthday week proportions (68,849/564,725, 12.19% vs 48,127/408,685, 11.78%; *χ*^2^_1_=153.25, *P*<.001).

#### Stratified by Months of Birth

**Figure 2.**
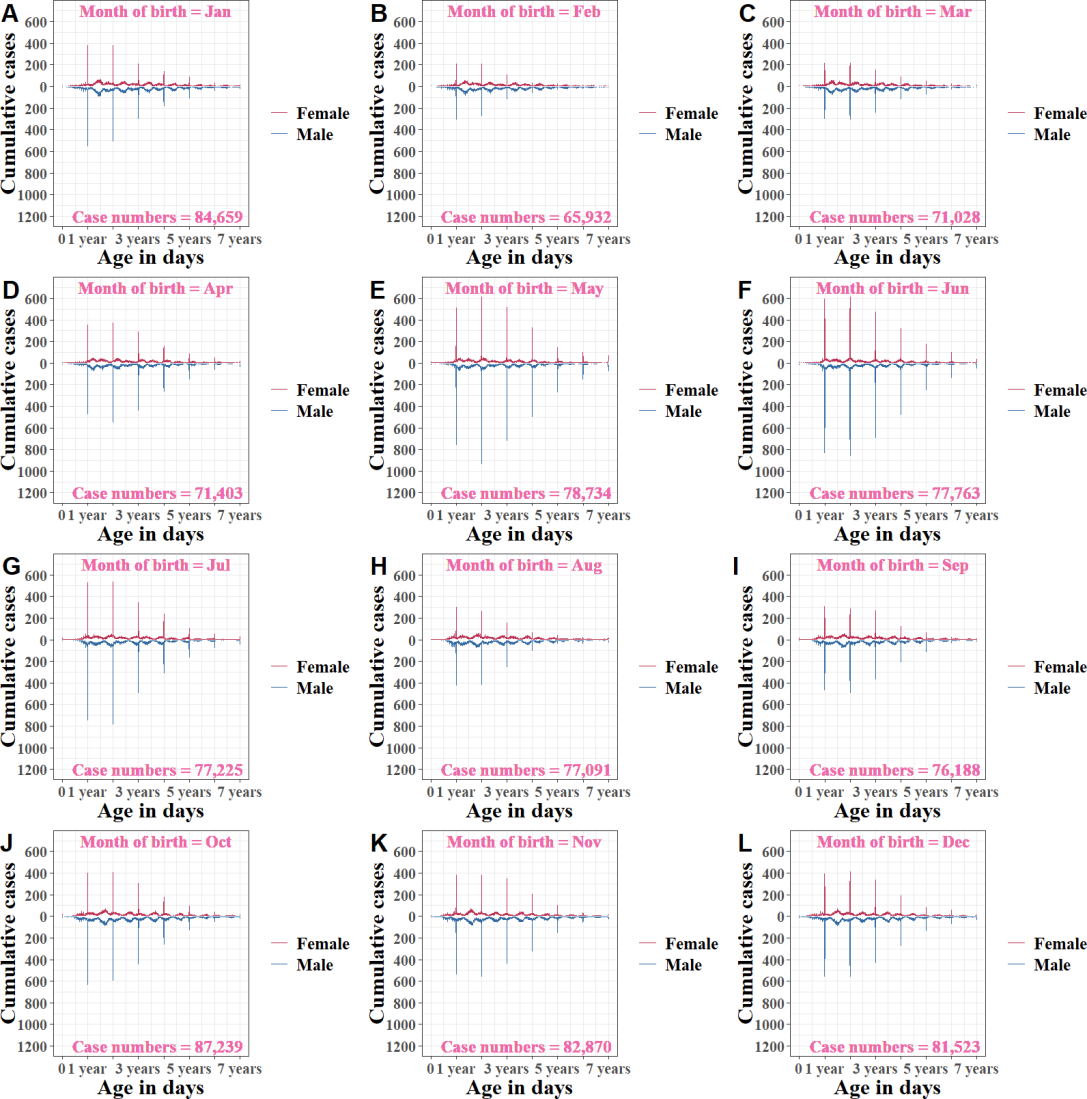
Cumulative HFMD cases by age (measured in days) and sex among children aged 0‐7 years, stratified by months of birth (A-L) in Yunnan Province, China (2008‐2022). HFMD: hand, foot, and mouth disease.

**Figure 3.**
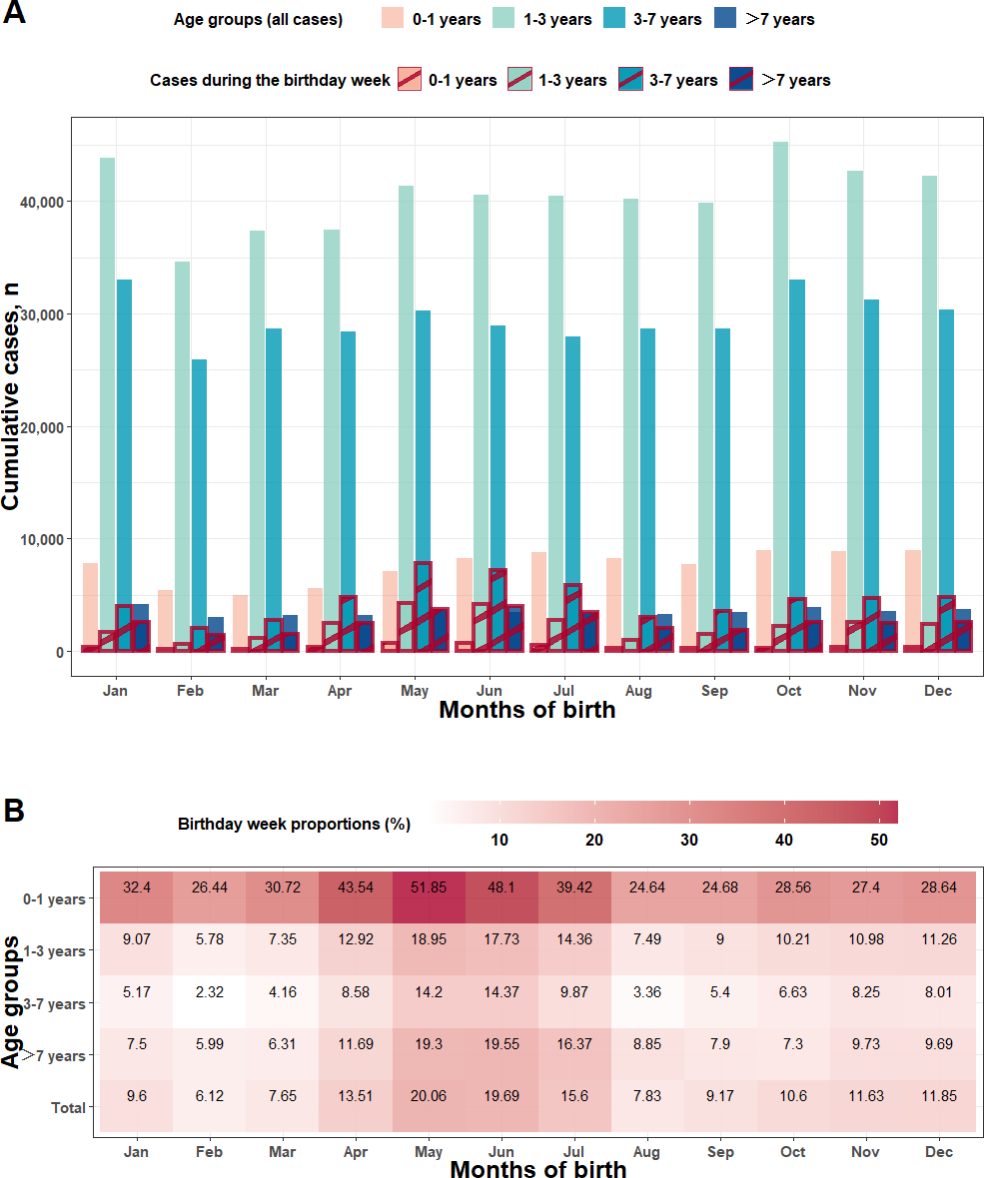
(A) Bar graph of cumulative HFMD cases and cases during the birthday week, and (B) heat map of birthday week proportions, across different age groups, stratified by months of birth in Yunnan Province, China (2008‐2022). HFMD: hand, foot, and mouth disease.

Analyses across age groups stratified by months of birth (Figure 2, Figure 3, and Table S2 in Multimedia Appendix 1) illustrated that there were differences in birthday week proportions among different months of birth (*χ*^2^_11_=16,942, *P*<.001). Patients born during the main peak from April to July had the highest birthday week proportions, with overall birthday week proportions of 13.51% (10,080/74,610), 20.26% (16,517/82,320), 19.69% (15,994/81,211), and 15.6% (12,568/80,543), respectively. Among them, the infant group (0‐1 y) had the highest birthday week proportions from April to July compared to other age groups, with values of 43.54% (2436/5595), 51.85% (3689/7115), 48.1% (3971/8256), and 39.42% (3450/8753), respectively. During the second peak from October to December, the overall birthday week proportions were 10.6% (9660/91,169), 11.63% (10,046/86,375), and 11.85% (10,102/85,219), respectively, and birthday week proportions in the infant group were 28.56% (2564/8979), 27.4% (2439/8901), and 28.64% (2557/8929), respectively. In the trough periods from January to March and from August to September, the overall birthday week proportions were lower than those in peak periods.

#### Stratified by Reporting Years

**Figure 4.**
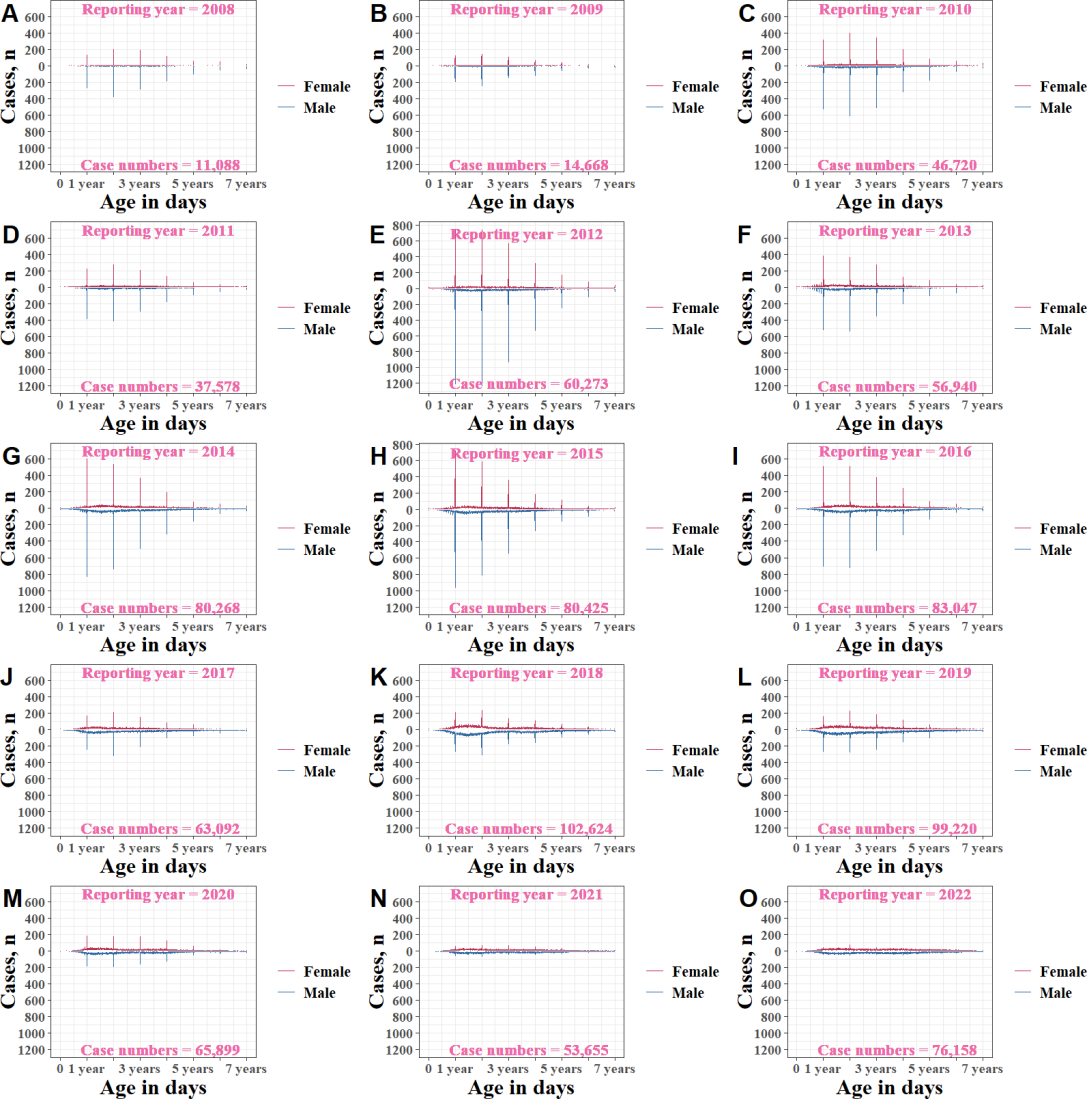
HFMD cases by age (measured in days) and sex among children aged 0‐7 years, stratified by reporting years (2008‐2022; A-O) in Yunnan Province, China. HFMD: hand, foot, and mouth disease.

**Figure 5.**
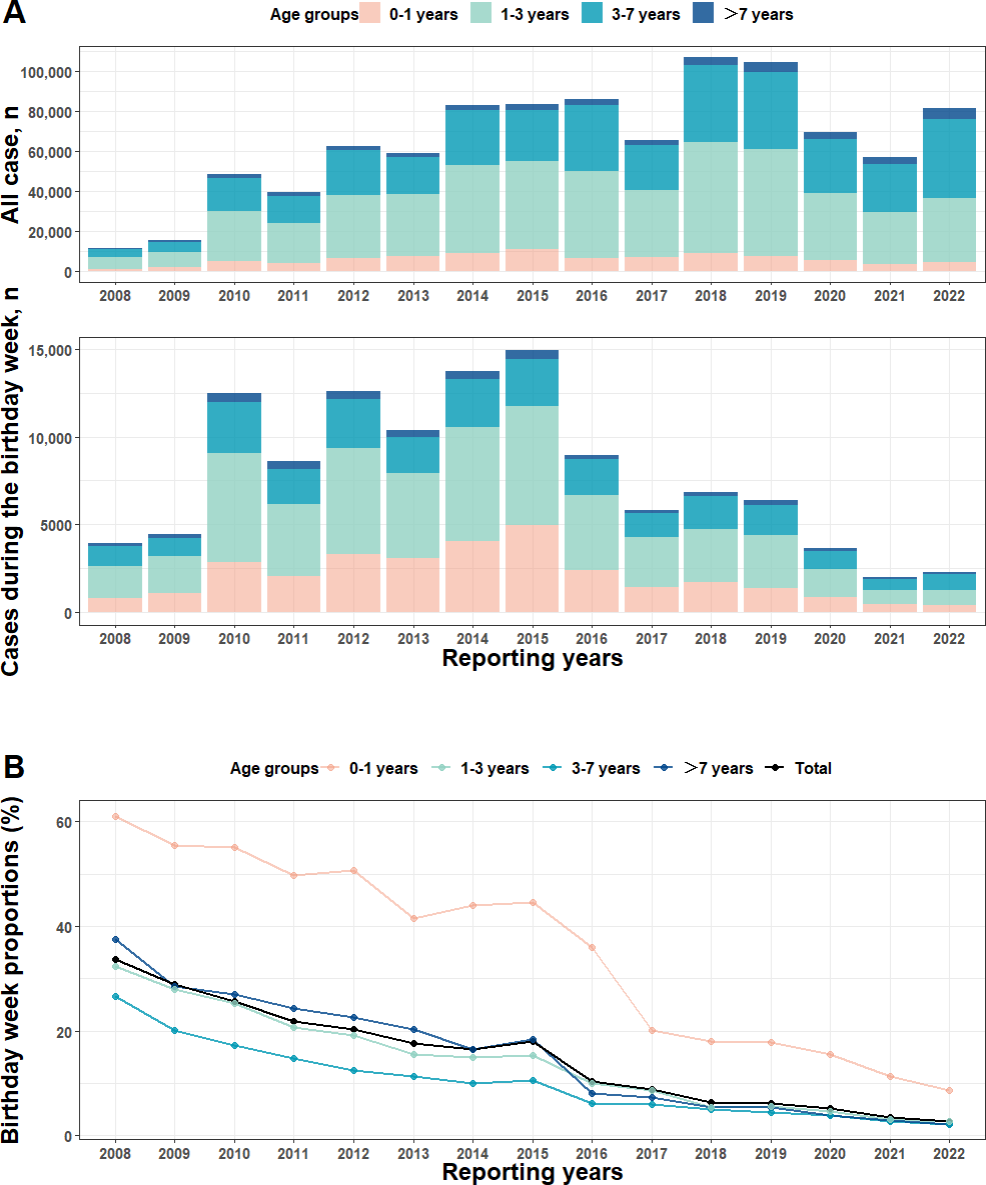
(A) The age group composition of cumulative HFMD cases and cases during the birthday week, and (B) birthday week proportions, with reporting years (2008‐2022) in Yunnan Province, China. HFMD: hand, foot, and mouth disease.

By conducting analyses across age groups stratified by reporting years (Figure 4, Figure 5, and Table S3 in Multimedia Appendix 1), we found a declining trend of overall birthday week proportions from 33.74% (3914/11,600) in 2008 to 2.77% (2254/81,372) in 2022 (Cochran-Armitage trend test: *Z*=−102.53, *P*<.001). This trend was observed across 4 different age groups, from 61.06% (784/1284) to 8.65% (383/4428) in the 0‐1 year group, from 32.36% (1846/5704) to 2.71% (873/32,253) in the 1‐3 years group, from 26.63% (1092/4100) to 2.23% (881/39,477) in the 3‐7 years group, and from 37.5% (192/512) to 2.24% (117/5214) in the >7 years group (*Z*=−44.71, −69.79, −48.16, and −25.57, respectively, all *P*<.001). There was a substantial point of decline: the birthday week proportion in 2017 was approximately half of that in 2015.

### The Results of Modified Poisson Regression

The modified Poisson regression model further supported the results of the simple subgroup descriptions. Compared to children aged >7 years, infants aged 0‐1 year were more likely to be infected with HFMD during their birthday week (relative risk [RR] 1.182, 95% CI 1.177‐1.185; *P*<.001). Compared to January, those born during the peak months of the HFMD epidemic—April (RR 1.037, 95% CI 1.035‐1.040; *P*<.001), May (RR 1.087, 95% CI 1.084‐1.090; *P*<.001), June (RR 1.084, 95% CI 1.081-1.087; *P*<.001), July (RR 1.048, 95% CI 1.045-1.051; *P*<.001), October (RR 1.007, 95% CI 1.005-1.010; *P*<.001), November (RR 1.017, 95% CI 1.014-1.019; *P*<.001), and December (RR 1.016, 95% CI 1.013-1.018; *P*<.001)—exhibited a higher propensity for contracting HFMD during their birthday week. Additionally, there was a decreasing trend in risks over the years, with a relatively significant decline from 2015 to 2017 (all *P*<.001; Table 2).

**Table 2.** Modified Poisson regression by conditional subgroups using HFMD^a^ surveillance data from Yunnan Province, China (2008‐2022).

Variables and subgroups		Relative risk	95% CI	*P* value
**Sex**				
	Female	1.000	0.998-1.001	.41
**Age group (years)**				
	0-1	1.182	1.177-1.185	<.001
	1-3	0.994	0.992-0.997	<.001
	3-7	0.968	0.965-0.970	<.001
**Month of birth**				
	Feb	0.972	0.970-0.975	<.001
	Mar	0.989	0.987-0.991	<.001
	Apr	1.037	1.035-1.040	<.001
	May	1.087	1.084-1.090	<.001
	Jun	1.084	1.081-1.087	<.001
	Jul	1.048	1.045-1.051	<.001
	Aug	0.982	0.980-0.985	<.001
	Sep	0.995	0.992-0.997	<.001
	Oct	1.007	1.005-1.010	<.001
	Nov	1.017	1.014-1.019	<.001
	Dec	1.016	1.013-1.018	<.001
**Reporting year**				
	2009	0.970	0.962-0.977	<.001
	2010	0.952	0.946-0.958	<.001
	2011	0.923	0.917-0.929	<.001
	2012	0.914	0.908-0.919	<.001
	2013	0.888	0.883-0.894	<.001
	2014	0.884	0.878-0.889	<.001
	2015	0.890	0.885-0.896	<.001
	2016	0.844	0.839-0.849	<.001
	2017	0.827	0.822-0.832	<.001
	2018	0.812	0.807-0.816	<.001
	2019	0.812	0.807-0.817	<.001
	2020	0.805	0.799-0.809	<.001
	2021	0.794	0.789-0.799	<.001
	2022	0.792	0.787-0.796	<.001

a HFMD: hand, foot, and mouth disease.

## Discussion

### Principal Findings

By analyzing the surveillance data of 973,410 HFMD cases in Yunnan Province, China, and presenting the age of cases by days, we found that 12.02% (n=116,976) of cases occurred during the birthday week, which is 6.27 times the average weekly proportion of 1.92% (7/365) in a year. Subgroup analyses demonstrated that the birthday week proportions were similar between male and female individuals. Significant differences in birthday week proportions among different age groups and months of birth were observed (all *P*<.001). Compared to other age groups, the infant group aged 0‐1 year had the highest birthday week proportions (30,539/90,709, 33.67%), which is 17.57 times the average weekly proportion. Compared to other months, patients born from April to July and from October to December, the peak months of the HFMD epidemic, had higher birthday week proportions. Additionally, there was a decreasing trend in birthday week proportions from 2008 to 2022, dropping from 33.74% (3914/11,600) to 2.77% (2254/81,372). The results of the modified Poisson regression model further supported the findings of these subgroup analyses.

Although the higher proportion of case numbers during the birthday week in infants compared to other age groups was partly due to the additive effect of the loss of maternal antibodies with increasing age in infancy [23,27], this combined result implied a relatively obvious practical significance for HFMD prevention and control. The larger birthday week proportions in those born during the peak months of the HFMD epidemic were due to the combined effect of the birthday week effect and the seasonality of HFMD. HFMD viruses are more prevalent in the natural environment during peak periods due to climatic and meteorological changes [13,14]. For the decreasing trends in birthday week proportions over the years, our assumption was that this might be related to the improvements in reporting quality due to the annual routine HFMD-related supervision, training, and assessment activities [28,29]. In the early years, the quality of reporting might not have been high. Notably, the significant drops from 2015 to 2017 coincided with events that likely improved reporting quality. From October 1, 2015, to September 30, 2016, to strengthen HFMD prevention and control in the country and improve surveillance, the General Office of the National Health and Family Planning Commission launched a pilot HFMD surveillance program, with Yunnan Province as one of the pilot provinces [30]. The improvements in reporting and surveillance might account for much of the decreasing trend in birthday week proportions. However, the heterogeneity in the decreasing rate among different age groups (eg, the birthday week proportions for the infant group aged 0‐1 y in 2022 was 14.1% [8.65%/61.06%] of that in 2008, whereas for the groups aged 1‐3 y and 3‐7 y, the birthday week proportions in 2022 were 8.37% [2.71%/32.36% and 2/23%/26.63%] of that in 2008, and for the group aged >7 y, it was 5.97% [2.24%/37.5%]) suggests that the phenomenon of the birthday week effect cannot not be completely explained by date-entry errors or inaccuracies in the data collection process. Additionally, analyzing the data in 2022 alone showed that all age groups had higher birthday week proportions than the average of 1.92% (7/365), especially the 0‐1 year infant group and 1‐3 years group, with 8.65% (383/4428) and 2.71% (873/32,253) of cases occurring during the birthday week, respectively. Furthermore, we noticed that the increased risk of HFMD spanned the week including the 6 days before the birthday, particularly the 3 days before, not just the exact birthday. This observation does not align well with the possible errors made by health care providers for convenience when entering patients’ information. Therefore, the birthday week effect phenomenon is indeed related to other factors.

The higher exposure risk may explain why the incidence of HFMD was higher during the patients’ birthday week. First, on the birthday, compared to other days of the year, family members, teachers, classmates, and friends might engage in celebration activities such as having meals together and going to playgrounds, which could increase the risk of exposure to and infection with HFMD [31]. It has been reported that birthday parties are associated with a higher risk of children contracting *Salmonella enteritidis* PT6 [32] and *Microsporum canis* [33]. Second, they might consume foods deviating from their usual diet, including birthday cakes, which could impact their gastrointestinal function and increase the risks of enterovirus infection [34,35].

Previous studies [15-21] that reported a positive correlation between birthdays and certain health and disease outcomes have also attributed their findings to birthday gatherings. For instance, birthday gatherings might serve as the distracting life events; patients who underwent surgery on a surgeon’s birthday exhibited higher mortality compared to those who had surgery on other days [18]. These studies also considered some psychosomatic mechanisms. For example, a birthday may represent an acute psychosocial stressor for some individuals, inducing various emotional, physical, and mental changes. Stressful life events can trigger vascular events such as acute stroke, myocardial infarction, and sudden cardiac death [19]. Additionally, some studies explained the birthday rhythm of diseases or death using concepts such as suicide [17] or “birthday blues” [16] (“birthday pressure”). The “postponement of death” phenomenon suggests that an approaching birthday or holiday might provide critically ill patients an extra incentive to live until that day [17]. The “birthday blues” phenomenon suggests that a birthday or holiday might remind individuals of traumatic experiences, causing anxiety and other negative feelings that could lead to suicide [16]. These mechanisms, however, do not seem to appropriately explain our findings. However, previous studies found that the higher risk occurred around the birthday [17,21], which is consistent with this study.

These mechanisms discussed above reveal some relatively apparent explanatory factors that have been widely discussed and partially accepted; however, they might not comprehensively explain our finding. We boldly hypothesize that the birthday week effect might also be related to the body’s biological rhythms. The birthday week effect appears to be synchronized with geophysical cycles. Many previous studies [36-38] have found that the circadian and circannual cycles of all organisms, including humans, are mainly regulated by their reaction to sunlight (such as photoperiod), and many of their behaviors and physiological activities (including immunity [39,40]) exhibit obvious rhythmicity in response to light. Presumably, experiencing a birthday is equivalent to experiencing a complete revolution cycle of Earth, with the same accumulated light and heat effects from the Earth’s revolution. However, the birthday week, situated at the junction of 2 accumulated cycles, might make individuals more susceptible to disease, possibly due to lowered immunity.

### Limitations

There were limitations to this study. We only revealed the phenomenon of the birthday week effect and provided possible explanations from several different angles; however, we lack certain experiments or practical evidence to verify these mechanisms. In the future, more studies are needed to investigate and explain this phenomenon. For example, research can be conducted to quantitatively assess the improvements in surveillance systems and reporting quality, as well as the exposure risks of HFMD during the birthday week. Additionally, data on other diseases could be analyzed to explore whether this phenomenon exists. Furthermore, related animal experiments could be carried out to verify possible biological mechanisms.

### Conclusion

In conclusion, this was an interesting study that reported a novel birthday week effect of HFMD, particularly for infants who were about to celebrate their first birthday and those born during the peak months of the HFMD epidemic. Improvements in surveillance systems and case reporting quality might explain the decreasing trend of the birthday week effect over the years. Additionally, high exposure risks during the birthday period might also account for the occurrence of this phenomenon. Although potential causes and physiological mechanisms need to be verified, our study provides theoretical evidence for the prevention and control of HFMD, especially for susceptible subgroups. To some extent, raising public awareness of the increased HFMD risk during this personally significant time and reducing unnecessary social activities during the birthday period will benefit HFMD prevention and control.

## Supplementary material

10.2196/59237 Multimedia Appendix 1 Cumulative hand, foot, and mouth disease (HFMD) cases per day and results stratified by months of birth and reporting years.

## Abbreviations

CDC: Centers for Disease Control and Prevention
HFMD: hand, foot, and mouth disease
NSNIDP: National Surveillance of Notifiable Infectious Disease Program
RR: relative risk

## References

[1] World Health Organization, Regional Office for the Western Pacific (2011). A Guide to Clinical Management and Public Health Response for Hand, Foot and Mouth Disease (HFMD).

[2] Li P, Huang Y, Zhu D, Yang S, Hu D (2021). Risk factors for severe hand-foot-mouth disease in China: a systematic review and meta-analysis. Front Pediatr.

[3] Koh WM, Badaruddin H, La H, Chen MIC, Cook AR (2018). Severity and burden of hand, foot and mouth disease in Asia: a modelling study. BMJ Glob Health.

[4] (2008). Circular of the Ministry of Health on the inclusion of HFMD in the management of notifiable infectious diseases [Article in Chinese]. The Central People’s Government of the People’s Republic of China.

[5] (2022). Overview of the epidemic of national statutory infectious diseases [Article in Chinese]. National Health Commission of the People’s Republic of China.

[6] Xing W, Liao Q, Viboud C (2014). Hand, foot, and mouth disease in China, 2008-12: an epidemiological study. Lancet Infect Dis.

[7] Puenpa J, Wanlapakorn N, Vongpunsawad S, Poovorawan Y (2019). The history of enterovirus A71 outbreaks and molecular epidemiology in the Asia-Pacific region. J Biomed Sci.

[8] Kaminska K, Martinetti G, Lucchini R, Kaya G, Mainetti C (2013). Coxsackievirus A6 and hand, foot and mouth disease: three case reports of familial child-to-immunocompetent adult transmission and a literature review. Case Rep Dermatol.

[9] Li J, Zhu R, Huo D (2018). An outbreak of coxsackievirus A6-associated hand, foot, and mouth disease in a kindergarten in Beijing in 2015. BMC Pediatr.

[10] Sittikul P, Batty EM, Yodsawat P (2023). Diversity of human enterovirus co-circulations in five kindergartens in Bangkok between July 2019 and January 2020. Viruses.

[11] Jiang L, Jiang H, Tian X, Xia X, Huang T (2021). Epidemiological characteristics of hand, foot, and mouth disease in Yunnan Province, China, 2008-2019. BMC Infect Dis.

[12] Peng D, Ma Y, Liu Y, Lv Q, Yin F (2020). Epidemiological and aetiological characteristics of hand, foot, and mouth disease in Sichuan Province, China, 2011-2017. Sci Rep.

[13] Xiao X, Gasparrini A, Huang J (2017). The exposure-response relationship between temperature and childhood hand, foot and mouth disease: a multicity study from mainland China. Environ Int.

[14] Abad FX, Pintó RM, Bosch A (1994). Survival of enteric viruses on environmental fomites. Appl Environ Microbiol.

[15] Lester D (2005). The “birthday blues” in a sample of Major League Baseball players’ suicides. Percept Mot Skills.

[16] Williams A, While D, Windfuhr K (2011). Birthday blues: examining the association between birthday and suicide in a national sample. Crisis.

[17] Peña PA (2015). A not so happy day after all: excess death rates on birthdays in the U.S. Soc Sci Med.

[18] Kato H, Jena AB, Tsugawa Y (2020). Patient mortality after surgery on the surgeon’s birthday: observational study. BMJ.

[19] Saposnik G, Baibergenova A, Dang J, Hachinski V (2006). Does a birthday predispose to vascular events?. Neurology (ECronicon).

[20] Kurup H, Uzoigwe CE (2017). The association between birthdays and medical emergencies. Int J Prev Med.

[21] Whaley CM, Cantor J, Pera M, Jena AB (2021). Assessing the association between social gatherings and COVID-19 risk using birthdays. JAMA Intern Med.

[22] Hong J, Liu F, Qi H (2022). Changing epidemiology of hand, foot, and mouth disease in China, 2013-2019: a population-based study. Lancet Reg Health West Pac.

[23] Zhou J, Zhou Y, Luo K (2022). The transfer of maternal antibodies and dynamics of maternal and natural infection-induced antibodies against coxsackievirus A16 in Chinese children 0-13 years of age: a longitudinal cohort study. BMC Med.

[24] Sripa B, Suwannatrai AT, Sayasone S, Do DT, Khieu V, Yang Y (2021). Current status of human liver fluke infections in the Greater Mekong Subregion. Acta Trop.

[25] (2022). China statistical yearbook [Article in Chinese]. Statistics Bureau of China.

[26] Noma H (2024). rqlm: R package for implementing the modified Poisson and least-squares regression analyses with simple commands. The Comprehensive R Archive Network.

[27] Wei X, Yang J, Gao L (2021). The transfer and decay of maternal antibodies against enterovirus A71, and dynamics of antibodies due to later natural infections in Chinese infants: a longitudinal, paired mother-neonate cohort study. Lancet Infect Dis.

[28] (2012). Strengthening prevention and treatment of hand, foot and mouth disease in Yunnan Province [Article in Chinese]. Health Commission of Yunnan Province.

[29] (2012). Notice of Yunnan Provincial Health Department on further strengthening prevention and control of hand, foot and mouth disease [Article in Chinese]. Health Commission of Yunnan Province.

[30] (2015). Notice on carrying out the pilot work of hand, foot and mouth disease surveillance [Article in Chinese]. National Health Commission of the People’s Republic of China.

[31] Li P, Li T, Gu Q (2016). Children’s caregivers and public playgrounds: potential reservoirs of infection of hand-foot-and-mouth disease. Sci Rep.

[32] Dodhia H, Kearney J, Warburton F (1998). A birthday party, home-made ice cream, and an outbreak of Salmonella enteritidis phage type 6 infection. Commun Dis Public Health.

[33] Subelj M, Marinko JS, Učakar V (2014). An outbreak of Microsporum canis in two elementary schools in a rural area around the capital city of Slovenia, 2012. Epidemiol Infect.

[34] Shen C, Xu Y, Ji J (2021). Intestinal microbiota has important effect on severity of hand foot and mouth disease in children. BMC Infect Dis.

[35] Guo X, Lan Z, Wen Y (2021). Synbiotics supplements lower the risk of hand, foot, and mouth disease in children, potentially by providing resistance to gut microbiota dysbiosis. Front Cell Infect Microbiol.

[36] Cassone VM, Yoshimura T, Scanes CG, Dridi S (2022). Sturkie’s Avian Physiology (Seventh Edition).

[37] Emery P, So WV, Kaneko M, Hall JC, Rosbash M (1998). CRY, a drosophila clock and light-regulated cryptochrome, is a major contributor to circadian rhythm resetting and photosensitivity. Cell.

[38] Rosbash M (2009). The implications of multiple circadian clock origins. PLoS Biol.

[39] Dopico XC, Evangelou M, Ferreira RC (2015). Widespread seasonal gene expression reveals annual differences in human immunity and physiology. Nat Commun.

[40] Onishi KG, Maneval AC, Cable EC (2020). Circadian and circannual timescales interact to generate seasonal changes in immune function. Brain Behav Immun.

[41] xinxinran01/birthday-week. GitHub.

