# Supplementary material for: Exploring the Birthday Week Effect on Hand, Foot, and Mouth Disease in Yunnan Province, China, From 2008 to 2022: Surveillance Data Analysis
Source: JMIR Public Health Surveill. 2024 Sep 9;10:e59237. doi: 10.2196/59237 (PMC11404391; doi:10.2196/59237)
Supplement: Multimedia Appendix 1 [file publichealth-v10-e59237-s001.docx]

**Supplementary Materials**

**Table S1 Cumulative HFMD cases proportion and ratios relative to the average daily proportion in everyday of 365 days in Yunnan Province, China (2008-2022)**

| The difference in days between onset date and last birthday | Cumulative cases (No.) | Proportion (%) | Times relative to the average daily proportion^a^ |
| --- | --- | --- | --- |
| **365(birthday)** | **18 341** | **1·88** | **6·88** |
| **364** | **41 930** | **4·31** | **15·72** |
| **363** | **25 945** | **2·67** | **9·73** |
| **362** | **14 982** | **1·54** | **5·62** |
| **361** | **8028** | **0·82** | **3·01** |
| **360** | **4442** | **0·46** | **1·67** |
| **359** | **3308** | **0·34** | **1·24** |
| **total of birthday week** | **116 976** | **12·02** | **6·27** |
| 358 | 2975 | 0·31 | 1·12 |
| 357 | 2632 | 0·27 | 0·99 |
| 356 | 2501 | 0·26 | 0·94 |
| 355 | 2340 | 0·24 | 0·88 |
| 354 | 2389 | 0·25 | 0·90 |
| 353 | 2309 | 0·24 | 0·87 |
| 352 | 2231 | 0·23 | 0·84 |
| 351 | 2351 | 0·24 | 0·88 |
| 358 | 2975 | 0·31 | 1·12 |
| 357 | 2632 | 0·27 | 0·99 |
| 356 | 2501 | 0·26 | 0·94 |
| 355 | 2340 | 0·24 | 0·88 |
| 354 | 2389 | 0·25 | 0·90 |
| 353 | 2309 | 0·24 | 0·87 |
| 352 | 2231 | 0·23 | 0·84 |
| 351 | 2351 | 0·24 | 0·88 |
| 350 | 2261 | 0·23 | 0·85 |
| 349 | 2414 | 0·25 | 0·91 |
| 348 | 2282 | 0·23 | 0·86 |
| 347 | 2294 | 0·24 | 0·86 |
| 346 | 2417 | 0·25 | 0·91 |
| 345 | 2279 | 0·23 | 0·85 |
| 344 | 2352 | 0·24 | 0·88 |
| 343 | 2427 | 0·25 | 0·91 |
| 342 | 2395 | 0·25 | 0·90 |
| 341 | 2297 | 0·24 | 0·86 |
| 340 | 2380 | 0·24 | 0·89 |
| 339 | 2310 | 0·24 | 0·87 |
| 338 | 2262 | 0·23 | 0·85 |
| 337 | 2435 | 0·25 | 0·91 |
| 336 | 2346 | 0·24 | 0·88 |
| 335 | 2632 | 0·27 | 0·99 |
| 334 | 2761 | 0·28 | 1·04 |
| 333 | 2893 | 0·3 | 1·08 |
| 332 | 2692 | 0·28 | 1·01 |
| 331 | 2466 | 0·25 | 0·92 |
| 330 | 2414 | 0·25 | 0·91 |
| 329 | 2428 | 0·25 | 0·91 |
| 328 | 2433 | 0·25 | 0·91 |
| 327 | 2255 | 0·23 | 0·85 |
| 326 | 2289 | 0·24 | 0·86 |
| 325 | 2273 | 0·23 | 0·85 |
| 324 | 2299 | 0·24 | 0·86 |
| 323 | 2236 | 0·23 | 0·84 |
| 322 | 2268 | 0·23 | 0·85 |
| 321 | 2312 | 0·24 | 0·87 |
| 320 | 2213 | 0·23 | 0·83 |
| 319 | 2282 | 0·23 | 0·86 |
| 318 | 2261 | 0·23 | 0·85 |
| 317 | 2286 | 0·23 | 0·86 |
| 316 | 2253 | 0·23 | 0·84 |
| 315 | 2297 | 0·24 | 0·86 |
| 314 | 2274 | 0·23 | 0·85 |
| 313 | 2295 | 0·24 | 0·86 |
| 312 | 2376 | 0·24 | 0·89 |
| 311 | 2316 | 0·24 | 0·87 |
| 310 | 2294 | 0·24 | 0·86 |
| 309 | 2188 | 0·22 | 0·82 |
| 308 | 2307 | 0·24 | 0·87 |
| 307 | 2244 | 0·23 | 0·84 |
| 306 | 2315 | 0·24 | 0·87 |
| 305 | 2391 | 0·25 | 0·90 |
| 304 | 2666 | 0·27 | 1·00 |
| 303 | 2775 | 0·29 | 1·04 |
| 302 | 2700 | 0·28 | 1·01 |
| 301 | 2544 | 0·26 | 0·95 |
| 300 | 2499 | 0·26 | 0·94 |
| 299 | 2428 | 0·25 | 0·91 |
| 298 | 2387 | 0·25 | 0·90 |
| 297 | 2299 | 0·24 | 0·86 |
| 296 | 2321 | 0·24 | 0·87 |
| 295 | 2281 | 0·23 | 0·86 |
| 294 | 2324 | 0·24 | 0·87 |
| 293 | 2323 | 0·24 | 0·87 |
| 292 | 2182 | 0·22 | 0·82 |
| 291 | 2312 | 0·24 | 0·87 |
| 290 | 2218 | 0·23 | 0·83 |
| 289 | 2239 | 0·23 | 0·84 |
| 288 | 2325 | 0·24 | 0·87 |
| 287 | 2228 | 0·23 | 0·84 |
| 286 | 2229 | 0·23 | 0·84 |
| 285 | 2332 | 0·24 | 0·87 |
| 284 | 2152 | 0·22 | 0·81 |
| 283 | 2326 | 0·24 | 0·87 |
| 282 | 2264 | 0·23 | 0·85 |
| 281 | 2173 | 0·22 | 0·81 |
| 280 | 2258 | 0·23 | 0·85 |
| 279 | 2371 | 0·24 | 0·89 |
| 278 | 2257 | 0·23 | 0·85 |
| 277 | 2368 | 0·24 | 0·89 |
| 276 | 2385 | 0·25 | 0·89 |
| 275 | 2255 | 0·23 | 0·85 |
| 274 | 2530 | 0·26 | 0·95 |
| 273 | 2584 | 0·27 | 0·97 |
| 272 | 2744 | 0·28 | 1·03 |
| 271 | 2489 | 0·26 | 0·93 |
| 270 | 2459 | 0·25 | 0·92 |
| 269 | 2433 | 0·25 | 0·91 |
| 268 | 2401 | 0·25 | 0·90 |
| 267 | 2354 | 0·24 | 0·88 |
| 266 | 2331 | 0·24 | 0·87 |
| 265 | 2241 | 0·23 | 0·84 |
| 264 | 2305 | 0·24 | 0·86 |
| 263 | 2265 | 0·23 | 0·85 |
| 262 | 2250 | 0·23 | 0·84 |
| 261 | 2294 | 0·24 | 0·86 |
| 260 | 2212 | 0·23 | 0·83 |
| 259 | 2378 | 0·24 | 0·89 |
| 258 | 2280 | 0·23 | 0·85 |
| 257 | 2214 | 0·23 | 0·83 |
| 256 | 2235 | 0·23 | 0·84 |
| 255 | 2296 | 0·24 | 0·86 |
| 254 | 2251 | 0·23 | 0·84 |
| 253 | 2340 | 0·24 | 0·88 |
| 252 | 2290 | 0·24 | 0·86 |
| 251 | 2220 | 0·23 | 0·83 |
| 250 | 2277 | 0·23 | 0·85 |
| 249 | 2346 | 0·24 | 0·88 |
| 248 | 2270 | 0·23 | 0·85 |
| 247 | 2401 | 0·25 | 0·90 |
| 246 | 2310 | 0·24 | 0·87 |
| 245 | 2393 | 0·25 | 0·90 |
| 244 | 2420 | 0·25 | 0·91 |
| 243 | 2632 | 0·27 | 0·99 |
| 242 | 2572 | 0·26 | 0·96 |
| 241 | 2666 | 0·27 | 1·00 |
| 240 | 2657 | 0·27 | 1·00 |
| 239 | 2552 | 0·26 | 0·96 |
| 238 | 2365 | 0·24 | 0·89 |
| 237 | 2449 | 0·25 | 0·92 |
| 236 | 2352 | 0·24 | 0·88 |
| 235 | 2257 | 0·23 | 0·85 |
| 234 | 2331 | 0·24 | 0·87 |
| 233 | 2349 | 0·24 | 0·88 |
| 232 | 2272 | 0·23 | 0·85 |
| 231 | 2390 | 0·25 | 0·90 |
| 230 | 2371 | 0·24 | 0·89 |
| 229 | 2306 | 0·24 | 0·86 |
| 228 | 2403 | 0·25 | 0·90 |
| 227 | 2253 | 0·23 | 0·84 |
| 226 | 2280 | 0·23 | 0·85 |
| 225 | 2310 | 0·24 | 0·87 |
| 224 | 2296 | 0·24 | 0·86 |
| 223 | 2328 | 0·24 | 0·87 |
| 222 | 2349 | 0·24 | 0·88 |
| 221 | 2358 | 0·24 | 0·88 |
| 220 | 2324 | 0·24 | 0·87 |
| 219 | 2330 | 0·24 | 0·87 |
| 218 | 2284 | 0·23 | 0·86 |
| 217 | 2228 | 0·23 | 0·84 |
| 216 | 2492 | 0·26 | 0·93 |
| 215 | 2313 | 0·24 | 0·87 |
| 214 | 2375 | 0·24 | 0·89 |
| 213 | 2517 | 0·26 | 0·94 |
| 212 | 2608 | 0·27 | 0·98 |
| 211 | 2580 | 0·27 | 0·97 |
| 210 | 2581 | 0·27 | 0·97 |
| 209 | 2609 | 0·27 | 0·98 |
| 208 | 2467 | 0·25 | 0·93 |
| 207 | 2422 | 0·25 | 0·91 |
| 206 | 2368 | 0·24 | 0·89 |
| 205 | 2332 | 0·24 | 0·87 |
| 204 | 2397 | 0·25 | 0·90 |
| 203 | 2338 | 0·24 | 0·88 |
| 202 | 2340 | 0·24 | 0·88 |
| 201 | 2398 | 0·25 | 0·90 |
| 200 | 2290 | 0·24 | 0·86 |
| 199 | 2326 | 0·24 | 0·87 |
| 198 | 2315 | 0·24 | 0·87 |
| 197 | 2467 | 0·25 | 0·93 |
| 196 | 2336 | 0·24 | 0·88 |
| 195 | 2382 | 0·24 | 0·89 |
| 194 | 2349 | 0·24 | 0·88 |
| 193 | 2340 | 0·24 | 0·88 |
| 192 | 2344 | 0·24 | 0·88 |
| 191 | 2418 | 0·25 | 0·91 |
| 190 | 2314 | 0·24 | 0·87 |
| 189 | 2408 | 0·25 | 0·90 |
| 188 | 2302 | 0·24 | 0·86 |
| 187 | 2441 | 0·25 | 0·92 |
| 186 | 2399 | 0·25 | 0·90 |
| 185 | 2428 | 0·25 | 0·91 |
| 184 | 2531 | 0·26 | 0·95 |
| 183 | 2549 | 0·26 | 0·96 |
| 182 | 2719 | 0·28 | 1·02 |
| 181 | 2837 | 0·29 | 1·06 |
| 180 | 2956 | 0·3 | 1·11 |
| 179 | 2941 | 0·3 | 1·10 |
| 178 | 2724 | 0·28 | 1·02 |
| 177 | 2564 | 0·26 | 0·96 |
| 176 | 2413 | 0·25 | 0·90 |
| 175 | 2371 | 0·24 | 0·89 |
| 174 | 2387 | 0·25 | 0·90 |
| 173 | 2415 | 0·25 | 0·91 |
| 172 | 2430 | 0·25 | 0·91 |
| 171 | 2291 | 0·24 | 0·86 |
| 170 | 2325 | 0·24 | 0·87 |
| 169 | 2408 | 0·25 | 0·90 |
| 168 | 2375 | 0·24 | 0·89 |
| 167 | 2447 | 0·25 | 0·92 |
| 166 | 2365 | 0·24 | 0·89 |
| 165 | 2444 | 0·25 | 0·92 |
| 164 | 2422 | 0·25 | 0·91 |
| 163 | 2318 | 0·24 | 0·87 |
| 162 | 2351 | 0·24 | 0·88 |
| 161 | 2279 | 0·23 | 0·85 |
| 160 | 2386 | 0·25 | 0·89 |
| 159 | 2356 | 0·24 | 0·88 |
| 158 | 2240 | 0·23 | 0·84 |
| 157 | 2409 | 0·25 | 0·90 |
| 156 | 2417 | 0·25 | 0·91 |
| 155 | 2268 | 0·23 | 0·85 |
| 154 | 2373 | 0·24 | 0·89 |
| 153 | 2388 | 0·25 | 0·90 |
| 152 | 2510 | 0·26 | 0·94 |
| 151 | 2483 | 0·26 | 0·93 |
| 150 | 2612 | 0·27 | 0·98 |
| 149 | 2554 | 0·26 | 0·96 |
| 148 | 2400 | 0·25 | 0·90 |
| 147 | 2434 | 0·25 | 0·91 |
| 146 | 2340 | 0·24 | 0·88 |
| 145 | 2308 | 0·24 | 0·87 |
| 144 | 2390 | 0·25 | 0·90 |
| 143 | 2329 | 0·24 | 0·87 |
| 142 | 2415 | 0·25 | 0·91 |
| 141 | 2300 | 0·24 | 0·86 |
| 140 | 2291 | 0·24 | 0·86 |
| 139 | 2397 | 0·25 | 0·90 |
| 138 | 2324 | 0·24 | 0·87 |
| 137 | 2366 | 0·24 | 0·89 |
| 136 | 2255 | 0·23 | 0·85 |
| 135 | 2338 | 0·24 | 0·88 |
| 134 | 2344 | 0·24 | 0·88 |
| 133 | 2310 | 0·24 | 0·87 |
| 132 | 2372 | 0·24 | 0·89 |
| 131 | 2370 | 0·24 | 0·89 |
| 130 | 2314 | 0·24 | 0·87 |
| 129 | 2256 | 0·23 | 0·85 |
| 128 | 2326 | 0·24 | 0·87 |
| 127 | 2238 | 0·23 | 0·84 |
| 126 | 2367 | 0·24 | 0·89 |
| 125 | 2321 | 0·24 | 0·87 |
| 124 | 2403 | 0·25 | 0·90 |
| 123 | 2447 | 0·25 | 0·92 |
| 122 | 2439 | 0·25 | 0·91 |
| 121 | 2598 | 0·27 | 0·97 |
| 120 | 2604 | 0·27 | 0·98 |
| 119 | 2593 | 0·27 | 0·97 |
| 118 | 2425 | 0·25 | 0·91 |
| 117 | 2438 | 0·25 | 0·91 |
| 116 | 2352 | 0·24 | 0·88 |
| 115 | 2361 | 0·24 | 0·89 |
| 114 | 2393 | 0·25 | 0·90 |
| 113 | 2328 | 0·24 | 0·87 |
| 112 | 2378 | 0·24 | 0·89 |
| 111 | 2353 | 0·24 | 0·88 |
| 110 | 2309 | 0·24 | 0·87 |
| 109 | 2337 | 0·24 | 0·88 |
| 108 | 2278 | 0·23 | 0·85 |
| 107 | 2411 | 0·25 | 0·90 |
| 106 | 2339 | 0·24 | 0·88 |
| 105 | 2332 | 0·24 | 0·87 |
| 104 | 2316 | 0·24 | 0·87 |
| 103 | 2372 | 0·24 | 0·89 |
| 102 | 2380 | 0·24 | 0·89 |
| 101 | 2365 | 0·24 | 0·89 |
| 100 | 2282 | 0·23 | 0·86 |
| 99 | 2375 | 0·24 | 0·89 |
| 98 | 2320 | 0·24 | 0·87 |
| 97 | 2326 | 0·24 | 0·87 |
| 96 | 2347 | 0·24 | 0·88 |
| 95 | 2375 | 0·24 | 0·89 |
| 94 | 2396 | 0·25 | 0·90 |
| 93 | 2359 | 0·24 | 0·88 |
| 92 | 2426 | 0·25 | 0·91 |
| 91 | 2615 | 0·27 | 0·98 |
| 90 | 2616 | 0·27 | 0·98 |
| 89 | 2605 | 0·27 | 0·98 |
| 88 | 2510 | 0·26 | 0·94 |
| 87 | 2467 | 0·25 | 0·93 |
| 86 | 2327 | 0·24 | 0·87 |
| 85 | 2434 | 0·25 | 0·91 |
| 84 | 2316 | 0·24 | 0·87 |
| 83 | 2315 | 0·24 | 0·87 |
| 82 | 2340 | 0·24 | 0·88 |
| 81 | 2321 | 0·24 | 0·87 |
| 80 | 2306 | 0·24 | 0·86 |
| 79 | 2292 | 0·24 | 0·86 |
| 78 | 2372 | 0·24 | 0·89 |
| 77 | 2248 | 0·23 | 0·84 |
| 76 | 2332 | 0·24 | 0·87 |
| 75 | 2344 | 0·24 | 0·88 |
| 74 | 2250 | 0·23 | 0·84 |
| 73 | 2286 | 0·23 | 0·86 |
| 72 | 2326 | 0·24 | 0·87 |
| 71 | 2343 | 0·24 | 0·88 |
| 70 | 2369 | 0·24 | 0·89 |
| 69 | 2422 | 0·25 | 0·91 |
| 68 | 2369 | 0·24 | 0·89 |
| 67 | 2325 | 0·24 | 0·87 |
| 66 | 2368 | 0·24 | 0·89 |
| 65 | 2395 | 0·25 | 0·90 |
| 64 | 2413 | 0·25 | 0·90 |
| 63 | 2416 | 0·25 | 0·91 |
| 62 | 2399 | 0·25 | 0·90 |
| 61 | 2570 | 0·26 | 0·96 |
| 60 | 2672 | 0·27 | 1·00 |
| 59 | 2662 | 0·27 | 1·00 |
| 58 | 2465 | 0·25 | 0·92 |
| 57 | 2436 | 0·25 | 0·91 |
| 56 | 2398 | 0·25 | 0·90 |
| 55 | 2233 | 0·23 | 0·84 |
| 54 | 2345 | 0·24 | 0·88 |
| 53 | 2305 | 0·24 | 0·86 |
| 52 | 2452 | 0·25 | 0·92 |
| 51 | 2420 | 0·25 | 0·91 |
| 50 | 2243 | 0·23 | 0·84 |
| 49 | 2378 | 0·24 | 0·89 |
| 48 | 2363 | 0·24 | 0·89 |
| 47 | 2277 | 0·23 | 0·85 |
| 46 | 2393 | 0·25 | 0·90 |
| 45 | 2410 | 0·25 | 0·90 |
| 44 | 2371 | 0·24 | 0·89 |
| 43 | 2309 | 0·24 | 0·87 |
| 42 | 2366 | 0·24 | 0·89 |
| 41 | 2305 | 0·24 | 0·86 |
| 40 | 2341 | 0·24 | 0·88 |
| 39 | 2384 | 0·24 | 0·89 |
| 38 | 2421 | 0·25 | 0·91 |
| 37 | 2326 | 0·24 | 0·87 |
| 36 | 2345 | 0·24 | 0·88 |
| 35 | 2398 | 0·25 | 0·90 |
| 34 | 2468 | 0·25 | 0·93 |
| 33 | 2313 | 0·24 | 0·87 |
| 32 | 2482 | 0·25 | 0·93 |
| 31 | 2416 | 0·25 | 0·91 |
| 30 | 2603 | 0·27 | 0·98 |
| 29 | 2704 | 0·28 | 1·01 |
| 28 | 2607 | 0·27 | 0·98 |
| 27 | 2533 | 0·26 | 0·95 |
| 26 | 2356 | 0·24 | 0·88 |
| 25 | 2465 | 0·25 | 0·92 |
| 24 | 2379 | 0·24 | 0·89 |
| 23 | 2445 | 0·25 | 0·92 |
| 22 | 2408 | 0·25 | 0·90 |
| 21 | 2349 | 0·24 | 0·88 |
| 20 | 2448 | 0·25 | 0·92 |
| 19 | 2404 | 0·25 | 0·90 |
| 18 | 2390 | 0·25 | 0·90 |
| 17 | 2380 | 0·24 | 0·89 |
| 16 | 2358 | 0·24 | 0·88 |
| 15 | 2370 | 0·24 | 0·89 |
| 14 | 2292 | 0·24 | 0·86 |
| 13 | 2361 | 0·24 | 0·89 |
| 12 | 2477 | 0·25 | 0·93 |
| 11 | 2446 | 0·25 | 0·92 |
| 10 | 2409 | 0·25 | 0·90 |
| 9 | 2403 | 0·25 | 0·90 |
| 8 | 2450 | 0·25 | 0·92 |
| 7 | 2552 | 0·26 | 0·96 |
| 6 | 2469 | 0·25 | 0·93 |
| 5 | 2519 | 0·26 | 0·94 |
| 4 | 2586 | 0·27 | 0·97 |
| 3 | 2614 | 0·27 | 0·98 |
| 2 | 2690 | 0·28 | 1·01 |
| 1 | 2923 | 0·3 | 1·10 |

Notes:(a) A year is calculated as 365 days, and the average daily proportion is about 0·27% (1/365).

**Table S2 Cumulative HFMD cases, cases during the birthday week, and birthday week proportions of different age groups, stratified by months of birth, in Yunnan Province, China (2008-2022)**

| Age groups | Jan.^a^ | Feb.^a^ | Mar.^a^ | Apr.^b^ | May.^b^ | Jun.^b^ | Jul.^b^ | Aug.^a^ | Sept.^a^ | Oct.^b^ | Nov.^b^ | Dec.^b^ | Total |
| --- | --- | --- | --- | --- | --- | --- | --- | --- | --- | --- | --- | --- | --- |
| **All cases(No·)** |  |  |  |  |  |  |  |  |  |  |  |  |  |
| (0 1] | 7808 | 5439 | 5000 | 5595 | 7115 | 8256 | 8753 | 8240 | 7694 | 8979 | 8901 | 8929 | 90 709 |
| (1 3] | 43 850 | 34 583 | 37 391 | 37 399 | 41 373 | 40 548 | 40 498 | 40 191 | 39 839 | 45 220 | 42 722 | 42 257 | 485 871 |
| (3 7] | 33 001 | 25 910 | 28 637 | 28 409 | 30 246 | 28 959 | 27 974 | 28 660 | 28 655 | 33 040 | 31 247 | 30 337 | 355 075 |
| ＞7 | 4135 | 3022 | 3187 | 3207 | 3586 | 3448 | 3318 | 3266 | 3455 | 3930 | 3505 | 3696 | 41 755 |
| Total | 88 794 | 68 954 | 74 215 | 74 610 | 82 320 | 81 211 | 80 543 | 80 357 | 79 643 | 91 169 | 86 375 | 85 219 | 973 410 |
| **Cases during the birthday week (No.)** |  |  |  |  |  |  |  |  |  |  |  |  |  |
| (0 1] | 2530 | 1438 | 1536 | 2436 | 3689 | 3971 | 3450 | 2030 | 1899 | 2564 | 2439 | 2557 | 30 539 |
| (1 3] | 3977 | 1998 | 2747 | 4831 | 7842 | 7189 | 5815 | 3010 | 3584 | 4618 | 4689 | 4757 | 55 057 |
| (3 7] | 1706 | 602 | 1190 | 2438 | 4294 | 4160 | 2760 | 962 | 1546 | 2191 | 2577 | 2430 | 26 856 |
| ＞7 | 310 | 181 | 201 | 375 | 692 | 674 | 543 | 289 | 273 | 287 | 341 | 358 | 4524 |
| Total | 8523 | 4219 | 5674 | 10 080 | 16 517 | 15 994 | 12 568 | 6291 | 7302 | 9660 | 10 046 | 10 102 | 116 976 |
| **Birthday Week Proportions (%)** |  |  |  |  |  |  |  |  |  |  |  |  |  |
| (0 1] | 32·40 | 26·44 | 30·72 | 43·54 | 51·85 | 48·10 | 39·42 | 24·64 | 24·68 | 28·56 | 27·40 | 28·64 | 33·67 |
| (1 3] | 9·07 | 5·78 | 7·35 | 12·92 | 18·95 | 17·73 | 14·36 | 7·49 | 9·00 | 10·21 | 10·98 | 11·26 | 11·33 |
| (3 7] | 5·17 | 2·32 | 4·16 | 8·58 | 14·20 | 14·37 | 9·87 | 3·36 | 5·40 | 6·63 | 8·25 | 8·01 | 7·56 |
| ＞7 | 7·50 | 5·99 | 6·31 | 11·69 | 19·30 | 19·55 | 16·37 | 8·85 | 7·90 | 7·30 | 9·73 | 9·69 | 10·83 |
| Total | 9·60 | 6·12 | 7·65 | 13·51 | 20·06 | 19·69 | 15·60 | 7·83 | 9·17 | 10·60 | 11·63 | 11·85 | 12·02 |

Notes: (a) January-March and August-September are the trough months of HFMD epidemic.

(b) April-July and October-December are the peak months of HFMD epidemic.

**Table S3 HFMD Case numbers, cases during the birthday week, and birthday week proportions by age groups, stratified by reporting years, in Yunnan Province, China**

| Age groups | 2008 | 2009 | 2010 | 2011 | 2012 | 2013 | 2014 | 2015 | 2016 | 2017 | 2018 | 2019 | 2020 | 2021 | 2022 | Total |
| --- | --- | --- | --- | --- | --- | --- | --- | --- | --- | --- | --- | --- | --- | --- | --- | --- |
| **All cases (No.)** |  |  |  |  |  |  |  |  |  |  |  |  |  |  |  |  |
| (0 1] | 1284 | 1883 | 5130 | 4141 | 6500 | 7320 | 9204 | 11 132 | 6618 | 6903 | 9244 | 7709 | 5543 | 3670 | 4428 | 90 709 |
| (1 3] | 5704 | 7683 | 24 724 | 19 919 | 31 653 | 31 392 | 43 551 | 44 021 | 43 365 | 33 554 | 55 426 | 53 273 | 33 629 | 25 724 | 32253 | 485 871 |
| (3 7] | 4100 | 5102 | 16 866 | 13 518 | 22 120 | 18 228 | 27 513 | 25 272 | 33 064 | 22 635 | 37 954 | 38 238 | 26 727 | 24 261 | 39477 | 355 075 |
| ＞7 | 512 | 693 | 1934 | 1777 | 2213 | 2066 | 2821 | 2795 | 3056 | 2442 | 4346 | 5046 | 3644 | 3196 | 5214 | 41 755 |
| Total | 11 600 | 15 361 | 48 654 | 39 355 | 62 486 | 59 006 | 83 089 | 83 220 | 86 103 | 65 534 | 106 970 | 104 266 | 69 543 | 56 851 | 81 372 | 973 410 |
| **Cases during the birthday week (No.)** |  |  |  |  |  |  |  |  |  |  |  |  |  |  |  |  |
| (0 1] | 784 | 1044 | 2827 | 2058 | 3295 | 3038 | 4042 | 4971 | 2379 | 1393 | 1673 | 1375 | 859 | 418 | 383 | 30 539 |
| (1 3] | 1846 | 2154 | 6242 | 4104 | 6072 | 4887 | 6492 | 6786 | 4307 | 2877 | 3042 | 2996 | 1575 | 804 | 873 | 55 057 |
| (3 7] | 1092 | 1029 | 2920 | 1997 | 2762 | 2050 | 2755 | 2688 | 2022 | 1341 | 1892 | 1720 | 1044 | 663 | 881 | 26 856 |
| ＞7 | 192 | 198 | 522 | 432 | 502 | 419 | 463 | 516 | 244 | 178 | 237 | 272 | 139 | 93 | 117 | 4524 |
| Total | 3914 | 4425 | 12 511 | 8591 | 12 631 | 10 394 | 13 752 | 14 961 | 8952 | 5789 | 6844 | 6363 | 3617 | 1978 | 2254 | 116 976 |
| **Birthday week Proportions (%)** |  |  |  |  |  |  |  |  |  |  |  |  |  |  |  |  |
| (0 1] | 61·06 | 55·44 | 55·11 | 49·70 | 50·69 | 41·50 | 43·92 | 44·66 | 35·95 | 20·18 | 18·10 | 17·84 | 15·50 | 11·39 | 8·65 | 33·67 |
| (1 3] | 32·36 | 28·04 | 25·25 | 20·60 | 19·18 | 15·57 | 14·91 | 15·42 | 9·93 | 8·57 | 5·49 | 5·62 | 4·68 | 3·13 | 2·71 | 11·33 |
| (3 7] | 26·63 | 20·17 | 17·31 | 14·77 | 12·49 | 11·25 | 10·01 | 10·64 | 6·12 | 5·92 | 4·98 | 4·50 | 3·91 | 2·73 | 2·23 | 7·56 |
| ＞7 | 37·50 | 28·57 | 26·99 | 24·31 | 22·68 | 20·28 | 16·41 | 18·46 | 7·98 | 7·29 | 5·45 | 5·39 | 3·81 | 2·91 | 2·24 | 10·83 |
| Total | 33·74 | 28·81 | 25·71 | 21·83 | 20·21 | 17·62 | 16·55 | 17·98 | 10·40 | 8·83 | 6·40 | 6·10 | 5·20 | 3·48 | 2·77 | 12·02 |
